# Effectiveness and safety of Danshen injection on heart failure: Protocol for a systematic review and meta-analysis

**DOI:** 10.1097/MD.0000000000015636

**Published:** 2019-05-31

**Authors:** Tianhui Yuan, Yi Chen, Xiaoqi Zhou, Xueying Lin, Qingsong Zhang

**Affiliations:** a The First Affiliated Hospital of Guangzhou University of Chinese Medicine; b Guangzhou University of Chinese Medicine, Guangzhou; c Shenzhen Baoan Traditional Chinese Medicine Hospital Group, Guangdong Sheng, China.

**Keywords:** Danshen injection, heart failure, protocol

## Abstract

**Background:**

Danshen injection (DSI) is a traditional Chinese medicine preparation extracted from Danshen (Salvia miltiorrhiza), which has the functions of promoting blood circulation and removing blood stasis. Heart failure (HF) is a complex cardiovascular disease, always leading to frequent onset and hospitalization, decreased quality of life, increased mortality, etc. Many clinical studies demonstrate that DSI has a good treatment on HF. We will provide a protocol to evaluate the effectiveness and safety of DSI for HF.

**Methods:**

We will systematically search 3 English databases (PubMed, Excerpta Medica database [EMBASE], the Cochrane Central Register of Controlled Trials [Cochrane Library]) and 4 Chinese databases (Chinese National Knowledge Infrastructure [CNKI], Chinese VIP Information, Wanfang Database, and Chinese Biomedical Literature Database [CBM]) for randomised controlled trials (RCT) of DSI for HF. Left ventricular ejection fraction (LVEF), ejection fraction, left ventricular end diastolic dimension (LVEDD), and six-minute walk distance (SWD) will be set as the primary outcome measures. The secondary outcome measures will include NT-pro BNP, quality of life and adverse reaction. All data will be analysed by using Stata 14.0 software and TSA v0.9 software. We will use *I*^2^ test statistics to assess the heterogeneity of included studies, and Begg's funnel plots and Egger's test to assess publication bias. Methodological quality will be assessed through a Cochrane risk of bias tool for randomized controlled trials (RCTs).

**Result:**

This study will provide a high quality evidence for DSI on HF.

**Conclusion:**

This protocol will provide a reliable evidence to evaluate the effectiveness and safety of DSI on HF.

**Registration:**

PROS-PERO CRD42019125274.

## 1 Introduction

### 1.1 Description of the condition

Heart failure (HF), a complex clinical syndrome, is the terminal stage of many cardiac diseases.^[1]^ As a major clinical and public health problem worldwide, HF was reported to affect more than 20 million people. In China, there are more 5 million individuals diagnosed as HF, equating to a quarter of the world's.^[2,3]^ HF results from serious structural or functional abnormalities of the heart, due to injury to the myocardium from a variety of causes, including ischemic, and nonischemic etiologies.^[4]^ Resulting in severe deficits in cardiac output and ejection fraction (EF), HF mainly manifests as symptoms of pulmonary and peripheral edema, reduced exercise tolerance, dyspnea, and fatigue.^[5,6]^ Recurrent hospitalizations, multiple organ failure and high mortality rate are common as the disease progresses.^[7]^ It's reported that advanced HF causes a 50% 5-year mortality rate and is associated with more than one-third of cardiovascular deaths in the United States. Furthermore, the annual cost of treating HF in the United States exceeds $40 billion.^[8,9]^ At present, integrative medicine therapy, which combines traditional Chinese medicine (TCM) and western medicine, has shown more advantages in treating HF than using western medicine alone, in the aspects of increasing efficacy, regulating constitution, reducing of side effects of western medicine, and so on.^[10]^ TCM injection, including solution, emulsion, and powder extracted from Chinese herbal medicine, has provided a new administration route for HF patients.^[11]^

### 1.2 Description of the intervention

Danshen injection (DSI), extracted from the dried root and rhizome of traditional Chinese medicine Danshen (Salvia miltiorrhiza), has been widely used to treat cardiovascular disease (CVD) in China.^[11–13]^ Danshen has the effect of activating blood circulation and removing blood stasis. Recent pharmacological studies have shown that the active components of Danshen can significantly reduce blood viscosity, protect cardiovascular, and reduce myocardial hypoxia damage.^[14,15]^ Many clinical trials have shown that DSI could significantly reduce the ventricular hypertrophy index, improve the function of ventricular myocardium, and regulate the diastolic function of the ventricle.^[16,17]^ However, there has been no systematic reviews or meta-analyses that summarize the effect and safety of DSI on HF based on relevant RCTs. Therefore, this study aim to synthesized the efficacy and safety of DSI on HF, and provide sufficient evidence to support the clinical application of DSI.

## 2 Methods

### 2.1 Study registration

The protocol has been registered on the International Prospective Register of Systematic Reviews (PROSPERO) (registration number, CRD42019125274) basing on the Preferred Reporting Items for Systematic Reviews and Meta-Analyses Protocols (PRISMA-P) statement guidelines.

### 2.2 Eligibility criteria

#### 2.2.1 Research type

We will include the randomized controlled trials (RCTs) that evaluated the efficacy and safety of DSI on HF, regardless of blinding, publication status, or language.

#### 2.2.2 Participant type

Participants who meet the Diagnostic criteria for chronic HF^[18]^ will be included in the study, regardless of age, gender, or duration of disease. Primary disease can be coronary heart disease, hypertension, dilated cardiomyopathy, and rheumatic heart disease.

#### 2.2.3 Intervention measures

Intervention group will combine DSI with western medicine as intervention therapy. Control group will only include conventional western medicine as intervention measures.

#### 2.2.4 Outcome measures

The primary outcome measures will be as follows: left ventricular ejection fraction (LVEF), EF, left ventricular end diastolic dimension (LVEDD), and six-minute walk distance (SWD).

The secondary outcome measures will include NT-pro BNP, quality of life, and adverse reaction.

#### 2.2.5 Exclusion criteria

Studies will be excluded if the study is duplicated; studies did not provide the data needed for meta-analysis.

### 2.3 Data sources

#### 2.3.1 Electronic search

We will carry out a systematic search in such databases as Cochrane Library, PubMed, Embase, Chinese National Knowledge Infrastructure (CNKI), Chinese Scientific Journal Database (VIP), Chinese Biomedical and Medical Database (CBM), and Wanfang Database. The time span was from inception to February 10, 2019. The retrieval type will be “Danshen injection” or “Danshen” AND “heart failure” or “cardiac failure” or “CHF” (abbreviation of congestive HF). Chinese translation of the above terms will be used for the Chinese databases search. Endnote software 8.1 will be used to remove the duplicate articles.

#### 2.3.2 Additional search

We will make a further search for other potential articles basing on reference lists of the retrieved studies. Relevant paper journals will also be consulted for the articles that are not included in electronic databases.

### 2.4 Study selection and data extraction

Two reviewers will use Endnote software 8.1 to preliminarily read the abstract of the articles obtained in the databases mentioned above, in order to include the studies that meet the criteria. Then a second analysis will be carried out by scanning the full text to decide the final eligibility. Disagreements were being solved through discussion or the help of a third investigator. A Preferred Reporting Items for Systematic Reviews and Meta-Analyses (PRISMA) flow chart will be designed to illustrate the study selection procedure.

On the basis of self-developed data extraction form, the following data were extracted:

1. the basic information of the included trials (e.g., author, publication year and et al);
2. the basic information of the included participants (e.g., number of participants, gender, mean and standard deviation for age, and et al);
3. interventions of control group and treatment group;
4. random method;
5. outcome measures.

### 2.5 Data analysis

#### 2.5.1 Risk of bias in the included studies

Risk of bias for each included RCT will be evaluated by *Cochrane Handbook*, version 5.1.0. from several aspects including random sequence generation, allocation concealment, blinding of outcome assessments, incomplete outcome data and selective outcome reporting, and so on. The risk of bias in each RCT will be judged as low, high, or unclear. Two reviewers will independently evaluate the methodological quality of each included study, and discrepancies will be solved by discussion. The quality of the inclusions will be evaluated according to the modified Jadad rating scale.

#### 2.5.2 Statistical analysis

Meta-analysis and test sequential analysis (TSA) of the included studies will be carried out respectively by Statistical software (Stata 14.0 software and TSA v0.9 software). Mean difference (MD) and 95% confidence intervals (CIs) will be used to evaluate the continuous variables. As for dichotomous variables, rate ratio, and 95% CIs will be used to evaluate the extracted data.

#### 2.5.3 Addressing missing data

If any data necessary for evaluation is missed, we will contact the corresponding authors of articles through all the avenues we can get. In case that sufficient data cannot be obtained in this way, we will make an analysis of the available data and discuss possible impact of missing data.

#### 2.5.4 Data synthesis

All data will be analysed by using Stata 14.0. Based on the results of heterogeneity test, fixed-effect model analysis will be conducted if there exists small or medium heterogeneity (*I*^2^ < 50%); in contrast, a random-effect model analysis will be selected if heterogeneity is significant (*I*^2^ > 50%).

#### 2.5.5 Assessment of heterogeneity

*I*^2^ test statistics will be used to assess the heterogeneity of included studies. *I*^2^ < 25% means no significant heterogeneity, *I*^2^ = 25–50% represents considered moderate heterogeneity and *I*^2^ > 50% indicates strong heterogeneity. Fixed-effect model will be applied if the heterogeneity among trials is significant (*I*^2^ ≥ 50%). If *I*^2^ < 50%, representing low heterogeneity, random-effects model will be chosen.

#### 2.5.6 Assessment of publication bias

On condition that more than 10 RCTs are included, publication bias will be assessed by Begg's funnel plots and Egger's test. An asymmetrical funnel plot or a *P*-value of <0.1 on Egger's test will be considered to indicate the presence of publication bias. On the contrary, if the points turn out to be symmetrically distributed on either side of the funnel plot, it will indicate there is no publication bias.

#### 2.5.7 Subgroup analysis

Subgroup analyses will be performed in terms of age, interventions, controls, and population area, if heterogeneity is ≥50%, indicating that it's significant.

#### 2.5.8 Sensitivity analysis

When the heterogeneity is high, we plan to conduct sensitivity analyses based on study type, sample size, and methodological quality.

#### 2.5.9 Evidence synthesis

We plan to evaluate the quality of evidence of the included studies, according to guidelines of the GRADE (Grading of Recommendations, Assessment, Development, and Evaluation). The evidence quality will be rated by four levels: high, moderate, low, or very low.

#### 2.5.10 Ethics and dissemination

Ethical approval will not be applied for because the relevant data we extracted doesn’t involve any individual privacy. We plan to publish this study, which evaluate the effectiveness and safety of DSI on HF, in a peer-reviewed journal or conference presentations.

## 3 Discussion

Heart failure, is the most serious and advanced stage of various heart diseases, with high mortality and rehospitalization rates, with a syndrome characterized as peripheral edema, fluid retention, and breathlessness.^[19,20]^ Diuretics, ACEIs, beta blockers, and ARB are considered to be four commonly used drugs for HF, which could reduce cardiac load and maximize cardiac function.^[21]^ Although western medicine for HF has a positive therapeutic effect, it also has some serious side effects, such as hypotension, renal impairment, arrhythmia, etc.^[19]^ According to the literature, active ingredients in DSI can prevent myocardial fibrosis, cardiac hypertrophy, hemodynamic deterioration, and improved systolic and diastolic dysfunction to improve HF.^[22–24]^ Therefore, it is necessary to explore a protocol of DSI on HF with higher therapeutic effects and few side effects.

This will be the first systematic review and meta-analysis of the efficacy and safety of DSI on HF. This protocol will provide evidence of the efficacy and safety of DSI on HF. In addition to this, this will also indicate changes in clinical outcome measure, therapeutic effect, adverse reactions, and side effects. The systematic review will have the potential to aid clinicians in decision-making regarding DSI and to benefit patients with HF.

## Author contributions

Qingsong Zhang conceived the study idea. Tianhui Yuan were responsible for the design of this systematic review. Yi Chen contributed to the data analysis plan. Xiaoqi Zhou drafted the manuscript and Xueying Lin edited. All authors provided feedback and approved the final manuscript.

**Conceptualization:** Tianhui Yuan.

**Formal analysis:** Yi Chen.

**Methodology:** Xiaoqi Zhou.

**Software:** Xueying Lin.

**Writing – original draft:** Qingsong Zhang.

**Writing – review & editing:** Tianhui Yuan.
